# An atypical GdpP enzyme linking cyclic nucleotide metabolism to osmotic tolerance and gene regulation in *Mycoplasma bovis*

**DOI:** 10.3389/fmicb.2023.1250368

**Published:** 2023-11-30

**Authors:** Xifang Zhu, Eric Baranowski, Zhiyu Hao, Xixi Li, Gang Zhao, Yaqi Dong, Yingyu Chen, Changmin Hu, Huanchun Chen, Christine Citti, Aiping Wang, Aizhen Guo

**Affiliations:** ^1^School of Life Sciences, Zhengzhou University, Zhengzhou, China; ^2^Longhu Laboratory of Advanced Immunology, Zhengzhou, China; ^3^The State Key Laboratory of Agricultural Microbiology, College of Veterinary Medicine, Huazhong Agricultural University, Wuhan, China; ^4^IHAP, Université de Toulouse, INRAE, ENVT, Toulouse, France; ^5^Key Laboratory of Preventive Veterinary Medicine in Hubei Province, The Cooperative Innovation Center for Sustainable Pig Production, Wuhan, China; ^6^Key Laboratory of Development of Veterinary Diagnostic Products, Ministry of Agriculture of the People’s Republic of China, Wuhan, China; ^7^Hubei International Scientific and Technological Cooperation Base of Veterinary Epidemiology, International Research Center for Animal Disease, Ministry of Science and Technology of the People’s Republic of China, Wuhan, China

**Keywords:** *Mycoplasma bovis*, phosphodiesterase, GdpP, single-stranded DNase, cyclic dinucleotide, ppGpp, osmotic tolerance, gene regulation

## Abstract

Nucleotide second messengers play an important role in bacterial adaptation to environmental changes. Recent evidence suggests that some of these regulatory molecular pathways were conserved upon the degenerative evolution of the wall-less mycoplasmas. We have recently reported the occurrence of a phosphodiesterase (PDE) in the ruminant pathogen *Mycoplasma bovis*, which was involved in c-di-AMP metabolism. In the present study, we demonstrate that the genome of this mycoplasma species encodes a PDE of the GdpP family with atypical DHH domains. Characterization of *M. bovis* GdpP (MbovGdpP) revealed a multifunctional PDE with unusual nanoRNase and single-stranded DNase activities. The alarmone ppGpp was found unable to inhibit c-di-NMP degradation by MbovGdpP but efficiently blocked its nanoRNase activity. Remarkably, MbovGdpP was found critical for the osmotic tolerance of *M. bovis* under K^+^ and Na^+^ conditions. Transcriptomic analyses further revealed the biological importance of MbovGdpP in tRNA biosynthesis, pyruvate metabolism, and several steps in genetic information processing. This study is an important step in understanding the role of PDE and nucleotide second messengers in the biology of a minimal bacterial pathogen.

## 1 Introduction

Cyclic nucleotides are important signaling molecules in both prokaryotes and eukaryotes. These second messengers relay signals of extracellular messengers and thus participate in signal transduction. A newly discovered second messenger in bacteria is c-di-AMP (Romling, 2008; Witte et al., 2008), which regulates many cellular processes, including cell size, biofilm formation, potassium ion and carnitine uptake, as well as antibiotic resistance (Ye et al., 2014; Schuster et al., 2016; Fahmi et al., 2017; Whiteley et al., 2017). Intracellular homeostasis of c-di-AMP is thus crucial for maintaining normal bacterial physiology.

Diadenylate cyclases (DAC) and phosphodiesterases (PDE) are the main proteins involved in c-di-AMP metabolism. DAC are responsible for the synthesis of c-di-AMP through a condensation reaction involving two molecules of ATP or ADP (Bai et al., 2012; Muller et al., 2015; Rosenberg et al., 2015). Several PDE have been identified that catalyze the degradation of c-di-AMP in bacteria, and are generally divided into three protein groups: DhhP, PgpH and GdpP (Huynh and Woodward, 2016; Commichau et al., 2019; He et al., 2020). Originally described in *Borrelia burgdorferi*, DhhP proteins are widely distributed in bacteria. They are characterized by a DHH-DHHA1 domain and have been reported to convert c-di-AMP to phosphoadenylyl adenosine (pApA) or AMP in several species, including *Mycobacterium tuberculosis*, *Mycoplasma pneumonia, Staphylococcus aureus*, and *Streptococcus pneumonia* (Cron et al., 2011; Yang et al., 2014; Bowman et al., 2016; Blotz et al., 2017). PgpH proteins contain a His-Asp (HD) domain and have been found to bind c-di-AMP and convert this substrate into pApA in *Listeria monocytogenes* (Huynh et al., 2015). Finally, GdpP proteins are characterized by a PAS/GGDEF motif located upstream of the DHH-DHHA1 domain and possess both PDE and ATPase functions (Rao et al., 2010). This protein family has been found to convert c-di-AMP to pApA, and can slightly degrade ATP to ADP in *Bacillus subtilis*, *Enterococcus faecalis* and *S. aureus* (Rao et al., 2010; Bai et al., 2013; Wang et al., 2017).

Mycoplasmas are wall-less bacteria of the class *Mollicutes*, whose evolution is mainly characterized by genome downsizing (Citti and Blanchard, 2013). Despite a reduced coding capacity, several species are successful pathogens causing debilitating diseases in humans and a wide range of animals including cattle, swine, and avian hosts (Citti et al., 2010). The genome of these minimal, self-replicating bacteria has been used as a model system for the design of synthetic bacterial genomes and the exploration of essential functions in a minimal cell (Hutchison et al., 2016). Yet, nucleotide signaling transduction systems are poorly characterized in these atypical organisms. Recent evidence suggests that nucleotide second messengers, such as (p)ppGpp and c-di-AMP, may play important biological functions in several mycoplasma species including the small ruminant pathogen *Mycoplasma capricolum* and the human pathogen *Mycoplasma pneumoniae* (Glaser et al., 1981; Halbedel et al., 2007; Blotz et al., 2017). In a previous study with the ruminant pathogen *Mycoplasma bovis*, we identified several DHH proteins with PDE and/or nanoRNase activities (Zhu et al., 2020). In the present study, we have used *M. bovis* as a model organism to further analyze genes involved in c-di-AMP metabolism and characterized the enzymatic activity of a putative GdpP PDE encoded by CDS Mbov_0276 (MBOV_RS01380) in strain HB0801. Remarkably, MbovGdpP was found to be a multifunctional enzyme exhibiting a ssDNase activity in addition to the typical PDE function associated with bacterial GdpP and the nanoRNase activity discovered previously (Zhu et al., 2020). The phenotypic characterization of MbovGdpP knock-out mutant identified this protein as important for osmotic tolerance in this species. Finally, the regulatory role played by MbovGdpP was highlighted by transcriptomic analysis revealing an influence of this PDE in tRNA expression, pyruvate metabolism, and several steps in genetic information processing.

## 2 Materials and methods

### 2.1 Bacterial strains and culture conditions

Bacterial strains used in this study are listed in Supplementary Table S1. *M. bovis* HB0801 (GenBank sequence CP002058.1; NCBI Reference Sequence NC_018077.1) was grown in pleuropneumonia-like organism (PPLO) medium (BD Company, Sparks, MD, United States) with or without 100 μg/mL gentamicin, as previously described (Han et al., 2015). Mycoplasma titers were determined based on colony counts after 2 to 5 days of incubation at 37°C (Zhu et al., 2020). *Escherichia coli* DH5α and BL21 were grown in Luria Bertani (LB) broth medium with appropriate antibiotics.

### 2.2 DNA constructions and recombinant protein purification

Since mycoplasmas use UGA as a tryptophan codon, the MbovGdpP nucleotide sequence was modified by converting UGA codons into UGG to avoid premature translation stops in *E. coli*. The modified MbovGdpP nucleotide sequence was synthesized by the Beijing Tianyi Huiyuan Bioscience & Technology Inc. The synthetic gene was digested by *Nco*I and *Xho*I (Takara, Dalian, China), and then was cloned into the plasmid vector pET28b (+) using T4 DNA Ligase (Takara, Dalian, China) to generate plasmid pMbovGdpP (Supplementary Table S1). DNA constructions were validated by DNA sequencing before transformation into *E. coli* BL21 for protein expression. Recombinant proteins were purified with the nickel affinity chromatography method using 1 L of *E. coli* cultures (Supplementary Table S1) induced by 0.8 mM b-D-1-thiogalactopyranoside (IPTG) for 20 h at 16°C. Purified recombinant proteins were analyzed by SDS-PAGE, and their concentration was determined by using the BCA protein assay kit (Thermo Fisher Scientific, Waltham, MA, United States). Purified proteins were stored at −80°C.

### 2.3 Enzymatic activity assays

The ssDNA was synthesized by the Wuhan TsingKe Biological Technology Inc. with the sequence listed in Supplementary Table S1. For ssDNase assay, 5 μM recombinant proteins were incubated with ssDNA at 37°C overnight in 20 mM Tris-HCl (pH 7.0) containing 2.5 mM MnCl_2_. The reaction was stopped by boiling for 10 min. After centrifuging at 20,800 × *g*, samples were analyzed by electrophoresis on 12% polyacrylamide gels and stained with ethidium bromide before visualization with an imaging system (ProteinSimple, Santa Clara, CA, United States). For enzymatic degradation efficiency assays, 50 μM c-di-AMP, 50 μM pApA, and 10 μM ssDNA were used as substrates to measure PDE, nanoRNase, and ssDNase activities, respectively. The reaction mixtures were incubated from 0 to 240 min at 37°C and analyzed by HPLC, as previously described. Briefly, the soluble components were separated on an RP C18 column (250 × 4.6 mm id, 5 μm; Thermo Fisher Scientific, Waltham, MA, United States). The mobile phase consisted of 90% phosphate buffer (30 mM K_2_HPO_4_, 20 mM KH_2_PO_4_; pH 6.0) and 10% methanol at a flow rate of 1 mL/min. The wavelength of the UV detector was set up at 254 nm and the injection volume of the autosampler was 10 μL. For ssDNase activity, the incubation time was extended to 480 min, and reaction mixtures were analyzed by polyacrylamide gels. The conversion rate c-di-AMP was calculated by converting the corresponding HPLC peak regions to concentrations according to the standard curve.

### 2.4 ppGpp inhibition assay

To test the ability of ppGpp in inhibiting the MbovGdpP PDE activity, the reaction mixture containing 50 μM c-di-AMP was incubated with increasing concentrations of ppGpp (0 to 200 μM; Biolog, Bremen, Germany). The reaction products were analyzed by HPLC.

### 2.5 Osmotic tolerance

Tolerance to osmotic stress was carried out in axenic conditions. Briefly, *M. bovis* (10^4^ CFUs) was grown in PPLO medium containing increasing concentrations of KCl or NaCl (0 to 250 mM; Sinopharm, Shanghai, China). Mycoplasma titers were determined each 12 h incubation at 37°C.

### 2.6 RNA isolation and quantitative RT-PCR

Mycoplasma cells were harvested from 3 mL of PPLO cultures by centrifugation at 12,000 × *g* for 5 min. Total RNA was isolated from *M. bovis* pellets using the TRIzol reagent (Invitrogen Corporation, Carlsbad, CA, United States) (Flores-Valdez et al., 2018). RNA was stored at −80°C. Quantitative RT-PCR was performed as described (Zhu et al., 2021).

### 2.7 Transcriptome analysis of differential expressed genes

The expression profiles of differentially expressed genes of *M. bovis* were determined by RNA-sequencing (RNA-seq). The cDNA libraries were constructed by using TruSeq Stranded Total RNA with Ribo-Zero Gold (Illumina, San Diego, CA, United States) according to the manufacturer’s instructions. The quality and purity of RNA samples were determined with an Agilent Technologies 2100 Bioanalyzer (Agilent, Santa Clara, CA, United States). The RNA-seq library was sequenced on an Illumina HiSeq X10 sequencer (Illumina, San Diego, CA, United States) to generate paired-end (2 × 150 bp) reads. Raw reads generated during high-throughput sequencing were fastq format sequences. High-quality clean reads were generated by using Trimmomatic software to remove the linker and filtered out low-quality bases, N-bases or low-quality reads. Rockhooper2 was used to align clean reads to the *M. bovis* HB0801 genome (NCBI Reference Sequence NC_018077.1). Both *M. bovis* wild-type strain and mutant were set up for 3 biological replicates. Differentially expressed genes (DEGs) were defined by 1.5-fold change with a *p*-value <0.05, a commonly used cut-off value in transcriptomoic studies (Pletzer et al., 2020; Brochado et al., 2021; Tremblay et al., 2021). RNA-seq data (raw fastq files and read counts) are deposited in the Gene Expression Omnibus (GEO) repository under accession number GSE233141.

### 2.8 Bioinformatic analysis

Protein sequences alignments between RecJ and RecJ-like proteins were performed using National Center for Biotechnology Information (NCBI) Cobalt multiple alignment tool^1^ combined with ESPript 3.0 software.^2^

### 2.9 Statistical analysis

Statistical analyses were performed with SPSS software (SPSS, Inc., Chicago, IL, United States). The unpaired student’s *t*-test was used for the comparisons of two groups, while one-way ANOVA was used for multiple comparison. The differences were considered to be significant when *p*-value was lower than 0.05.

## 3 Results

### 3.1 Single-stranded DNase activity of MbovGdpP

**Figure 1:**
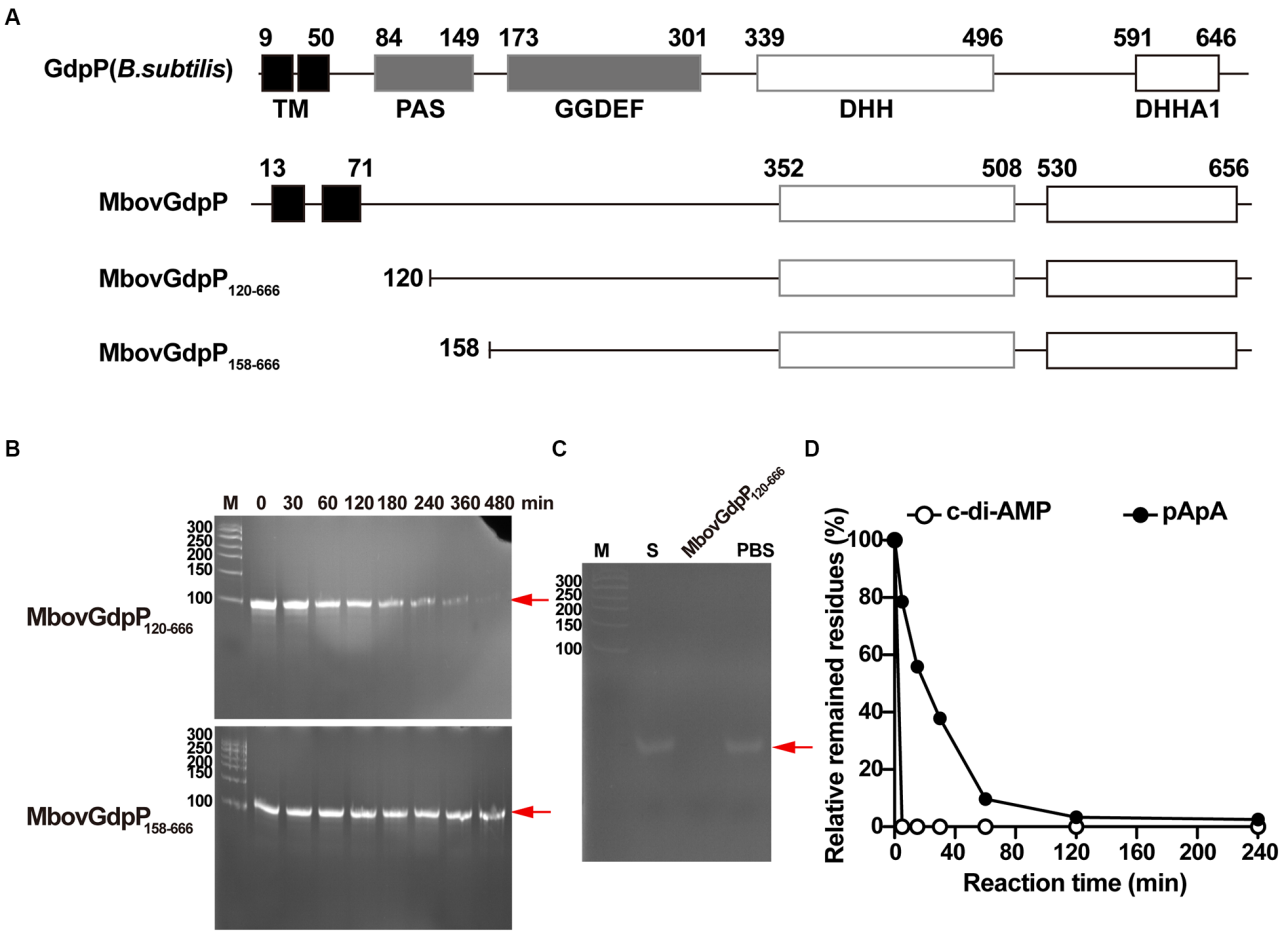
Enzymatic efficiency of MbovGdpP in degrading various substrates. **(A)** Schematic diagram of typical GdpP of *B. subtilis*, MbovGdpP, and truncated MbovGdpP_120–666_ and MbovGdpP_158–666_. In MbovGdpP_120–666_, the deleted region spans amino acids 1 to 119, while in MbovGdpP_158–666_, the deletion spans amino acids 1 to 157. **(B)** Polyacrylamide gel analysis of ssDNA degradation upon incubation with MbovGdpP_120–666_ (upper panel) and truncated MbovGdpP_158–666_ (lower panel). The red arrow indicates the position of the ssDNA following 0, 30, 60, 120, 180, 240, 360, and 480 min of incubation. The Lane M is the DNA ladder. **(C)** Using MbovGdpP_120–666_ as a control to analyze the catalyzation activity on ssDNA after 480 min. M: 50 bp DNA ladder; S: ssDNA in H_2_O; PBS: ssDNA in PBS. **(D)** HPLC analysis of c-di-AMP and pApA degradation by MbovGdpP_120–666_. The incubation time ranged from 0–240 min. The *y*-axis indicates the proportion of remaining c-di-AMP or pApA in the reacted system relative to initial concentration of c-di-AMP or pApA. The results displayed are from a typical experiment. The concentration of MbovGdpP_120–666_ and MbovGdpP_158–666_ used in present study were 5 μM, the concentration of ssDNA was 10 μM, while both c-di-AMP or pApA were 50 μM.

Unlike typical members of the GdpP family, such as *B. subtilis* GdpP, MbovGdpP is only characterized by a GGDEF-like motif and a DHH-DHHA1 domain, but no PAS motif (Figure 1). Yet, sequence comparison with other members of the DHH superfamily revealed similarity with prokaryotic subfamily I members including cyclic nucleotide phosphodiesterase, nanoRNases, and exonuclease RecJ (Srivastav et al., 2019; Wang et al., 2020). In particular, MbovGdpP sequence alignment with RecJ and RecJ-like proteins revealed an important level of similarity (Supplementary Figure S1). This led us to test the ssDNase activity of MbovGdpP with a synthetic oligonucleotide (Supplementary Table S1). The MbovGdpP_120–666_ and MbovGdpP_158–666_ were successfully expressed in *E. coli*, and the molecular weight of these proteins were 61.8 kDa and 57.0 kDa, respectively (Supplementary Figure S2). Polyacrylamide gel electrophoresis (PAGE) revealed a progressive degradation of the ssDNA concentration upon incubation with a recombinant MbovGdpP having the N-terminal transmembrane domain deleted amino acid residues 0 to 119 (MbovGdpP_120–666_) (Zhu et al., 2020), but not with the truncated MbovGdpP_158–666_ having the N-terminal region deleted up to amino acid 157 (Zhu et al., 2020) (Figures 1A–C). The catalytic efficiency of MbovGdpP on ssDNA was estimated to be less than 0.02 μM L^−1^ min^−1^, with a nearly complete degradation of ssDNA within 480 min. These data suggest that MbovGdpP has ssDNase activity and that amino-acid residues 120–158 are essential for MbovGdpP ssDNase activity.

### 3.2 c-di-AMP and pApA degradation by MbovGdpP

To further characterize MbovGdpP enzymatic activity, we have analyzed the degradation efficiency of c-di-AMP and pApA upon incubation with the recombinant protein MbovGdpP. Quantitative analyses revealed a complete degradation of c-di-AMP and pApA within 5 min and 120 min, respectively (Figure 1D). The catalytic rate of MbovGdpP on c-di-AMP and pApA was nearly 10 μM L^−1^ min^−1^ and 0.42 μM L^−1^ min^−1^, respectively. These results suggest that c-di-AMP and pApA may be the preferential substrates of MbovGdpP when compared to ssDNA.

### 3.3 NanoRNase but not phosphodiesterase activity of MbovGdpP is inhibited by ppGpp

**Figure 2:**
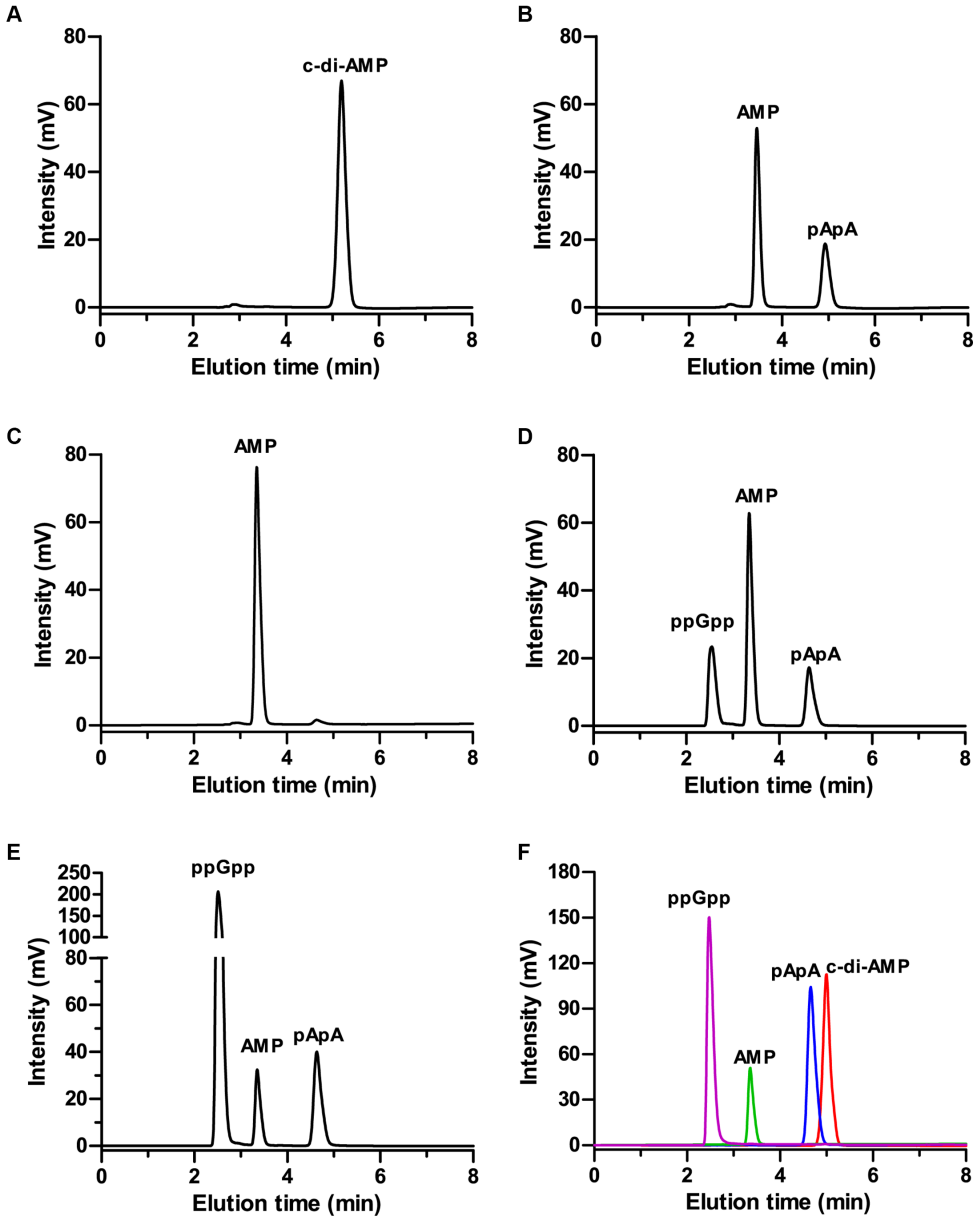
Inhibition of MbovGdpP enzymatic activities by ppGpp. **(A,B)** HPLC analysis of c-di-AMP degradation by MbovGdpP in 0 min **(A)** and 30 min **(B)**; **(C–E)** HPLC analysis of c-di-AMP degradation by MbovGdpP in the presence of 0 mM **(C)**, 50 mM **(D)** and 200 mM **(E)** of ppGpp. **(F)** Elution time of ppGpp (pink), c-di-AMP (red), pApA (blue) and AMP (green) standards. The concentration of MbovGdpP and c-di-AMP were 5 μM and 50 μM, respectively.

To test the capacity of ppGpp to inhibit MbovGdpP enzymatic activities, c-di-AMP degradation was analyzed following complete and incomplete reaction conditions. Data revealed that c-di-AMP degradation into AMP includes the formation of a pApA intermediate (Figures 2A,B). Remarkably, HPLC analysis only identified the accumulation of the pApA intermediate product upon c-di-AMP degradation in the presence of ppGpp, even after overnight incubation (Figures 2C–F). This result indicates that ppGpp is only able to inhibit the nanoRNase activity of MbovGdpP, but not its PDE activity. Altogether, these data highlight the unique enzymatic properties of MbovGdpP.

### 3.4 MbovGdpP enhances *Mycoplasma bovis* resistance to K^+^ stress

**Figure 3:**
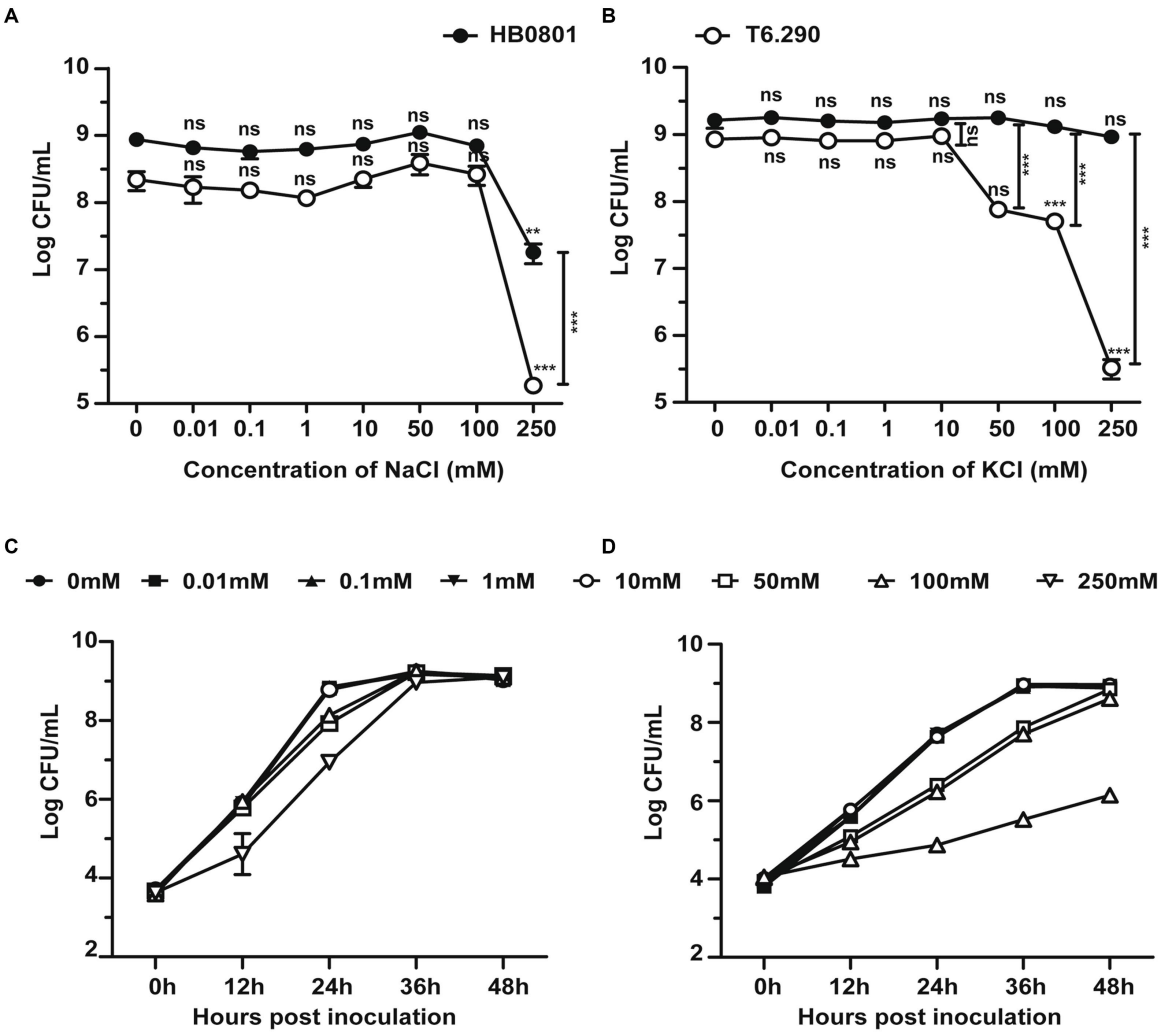
The osmotic tolerance of *M. bovis* is impaired in MbovGdpP knock-out mutants. Growth of MbovGdpP knock-out mutant (T6.290) and parental strain (HB0801) in PPLO medium in the presence of increasing concentration of Na^+^ **(A)** and K^+^ **(B)**. Mycoplasma titers were determined after 48 h of incubation. Growth curve of HB0801 **(C)** and T6.290 **(D)** in PPLO medium under different KCl concentrations. The data are presented as the means of three independent assays. Standard deviations are indicated by error bars. *p*-values are indicated by asterisks (^**^p < 0.01, ^***^p < 0.001, ns = *p* > 0.05).

Deletion of GdpP can alter bacterial resistance to osmotic stress (Pham et al., 2018; Teh et al., 2019). In the present study, *M. bovis* resistance to salt stress was tested by using the MbovGdpP knock-out mutant T6.290 (Zhu et al., 2020). When compare to wild-type strain HB0801, T6.290 growths in PPLO medium containing increasing concentrations of KCl/NaCl was found to be affected by KCl concentrations higher or equal to 50 mM, but not by NaCl (Figure 3). This result indicates that MbovGdpP may play important role in *M. bovis* resistance to potassium (K^+^) stress.

### 3.5 MbovGdpP plays an important role in tRNA biosynthesis and pyruvate metabolism

**Figure 4:**
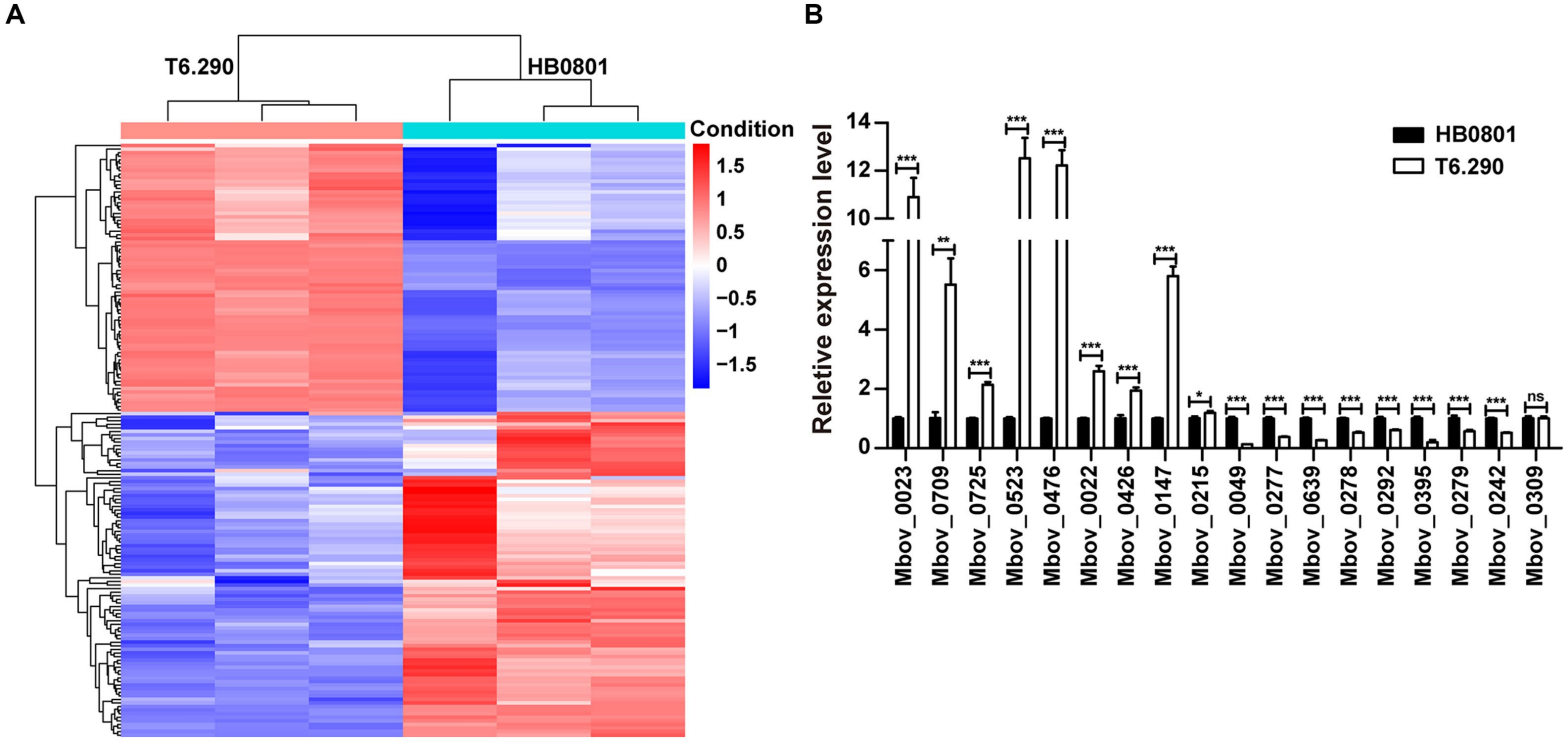
Differentially expressed transcriptome profiles of *M. bovis* HB0801 and mutant T6.290. **(A)** Heatmap showing DEGs (difference ≥1.5-folds; *p* < 0.05) between parental strain (HB0801) and the MbovGdpP knock-out mutant T6.290 (T6.290). For each strain, transcriptomic analyses were carried in triplicates. Each column represents a replicate, each row represents one gene. The red and blue color represent the relative up-regulated and down-regulated DEGs, respectively. The dendrogram at the top and left of the figure clusters the relationships of samples and genes, respectively. **(B)** qRT-PCR analyzing the relative expression level of DEGs expression in *M. bovis* parental strain (HB0801) and mutant T6.290 (T6.290). The data are presented as the means of three independent assays. Standard deviations are indicated by error bars. *p*-values are indicated by asterisks (^*^p < 0.05, ^**^p < 0.01, ^***^p < 0.001, ns = *p* > 0.05).

To better understand the role of MbovGdpP in cellular processes, RNA-seq was used to determine the transcriptional profile of T6.290 and HB0801 grown to the stationary phase. Differential gene expression analysis identified up to 161 genes with significant changes in T6.290, with 74 mRNA up-regulated and 87 down-regulated (Figure 4A and Supplementary Table S2). The accuracy of RNA-seq was further validated by qRT-PCR. Upregulated (*n* = 8) and down-regulated (*n* = 10) genes were selected among the most significant differentially expressed genes (DEGs) in T6.290. The qRT-PCR analysis confirmed changes in mRNA levels for 17 out of the 18 genes selected, including 9 up-regulated genes Mbov_0022 (deoxyguanosine kinase), Mbov_0023 (deoxyguanosine kinase), Mbov_0147 (hypothetical protein), Mbov_0215 (hypothetical protein), Mbov_0426 (glycine cleavage system protein H), Mbov_0476 (PTS sugar transporter subunit IIA), Mbov_0523 (DNA-binding protein WhiA), Mbov_0709 (DNA adenine methylase), Mbov_0725 (Cof-type HAD-IIB family hydrolase), and 8 down-regulated genes Mbov_0049 (hypothetical protein), Mbov_0242 (S8 family peptidase), Mbov_0277 (50S ribosomal protein L9), Mbov_0278 (replicative DNA helicase), Mbov_0279 (DUF21 domain-containing protein), Mbov_0292 (variable surface lipoprotein), Mbov_0395 (hypothetical protein), Mbov_0639 (hypothetical protein) (Figure 4B).

Interestingly, tRNA accounted for over 26% (23/87) of the down-regulated genes (Table 1), representing 67% of the total number of tRNA genes (23/34). The tRNA Arg, Gly, Ile, Leu, Lys, Met, Ser, Thr, and Trp are encoded by more than one gene. Among them, 7 tRNA genes (tRNA Arg, Leu, Lys, Met, Ser, Thr, and Trp) were down-regulated, while tRNA Gly and Ile remained unchanged. The remaining tRNAs (*n* = 9) were encoded by one gene, among them, six were differentially expressed in T6.290. These results indicate that MbovGdpP plays an important role in tRNA biosynthesis which may further influence gene expression in *M. bovis*.

**Table 1:** Differential expressed tRNA genes in *M. bovis* MbovGdpP knock-out mutant T6.290.

Gene	Product	Seq.	Position	Fold changes	*p*-value
Mbov_tRNA15	tRNA-Asn	gtt	301681–301683	0.512231	0.008801
Mbov_tRNA9	tRNA-Met	cat	289713–289715	0.503700	0.003385
Mbov_tRNA34	tRNA-Trp	cca	474372–474445	0.493298	0.031258
Mbov_tRNA29	tRNA-Thr	cgt	952332–952405	0.489691	0.002568
Mbov_tRNA17	tRNA-Val	tac	301808–301883	0.488978	0.012596
Mbov_tRNA18	tRNA-Thr	tgt	301886–301961	0.463227	0.002711
Mbov_tRNA14	tRNA-Phe	gaa	290143–290218	0.437130	0.013866
Mbov_tRNA27	tRNA-Leu	caa	971964–972047	0.429675	0.002130
Mbov_tRNA12	tRNA-Met	cat	289982–290057	0.423892	0.013597
Mbov_tRNA30	tRNA-Ser	cga	912775–912864	0.418897	0.015990
Mbov_tRNA16	tRNA-Glu	ttc	301729–301804	0.412727	0.001471
Mbov_tRNA19	tRNA-Leu	tag	301970–302054	0.410065	0.002163
Mbov_tRNA28	tRNA-Trp	tca	955330–955404	0.403808	0.004407
Mbov_tRNA13	tRNA-Asp	gtc	290062–290138	0.365602	0.017434
Mbov_tRNA26	tRNA-Thr	ggt	951789–951864	0.362123	0.000132
Mbov_tRNA25	tRNA-Lys	ctt	934711–934786	0.356144	0.007747
Mbov_tRNA22	tRNA-Ser	gct	627996–628088	0.354050	0.023577
Mbov_tRNA31	tRNA-His	gtg	888380–888455	0.312977	0.027021
Mbov_tRNA11	tRNA-Ser	tga	289859–289951	0.309675	0.001887
Mbov_tRNA5	tRNA-Arg	tct	85616–85692	0.286589	0.000341
Mbov_tRNA6	tRNA-Arg	cct	85759–85834	0.281319	0.002602
Mbov_tRNA2	tRNA-Leu	taa	3026–3100	0.276278	0.000535
Mbov_tRNA24	tRNA-Lys	ttt	801958–802033	0.268189	0.000199

**Figure 5:**
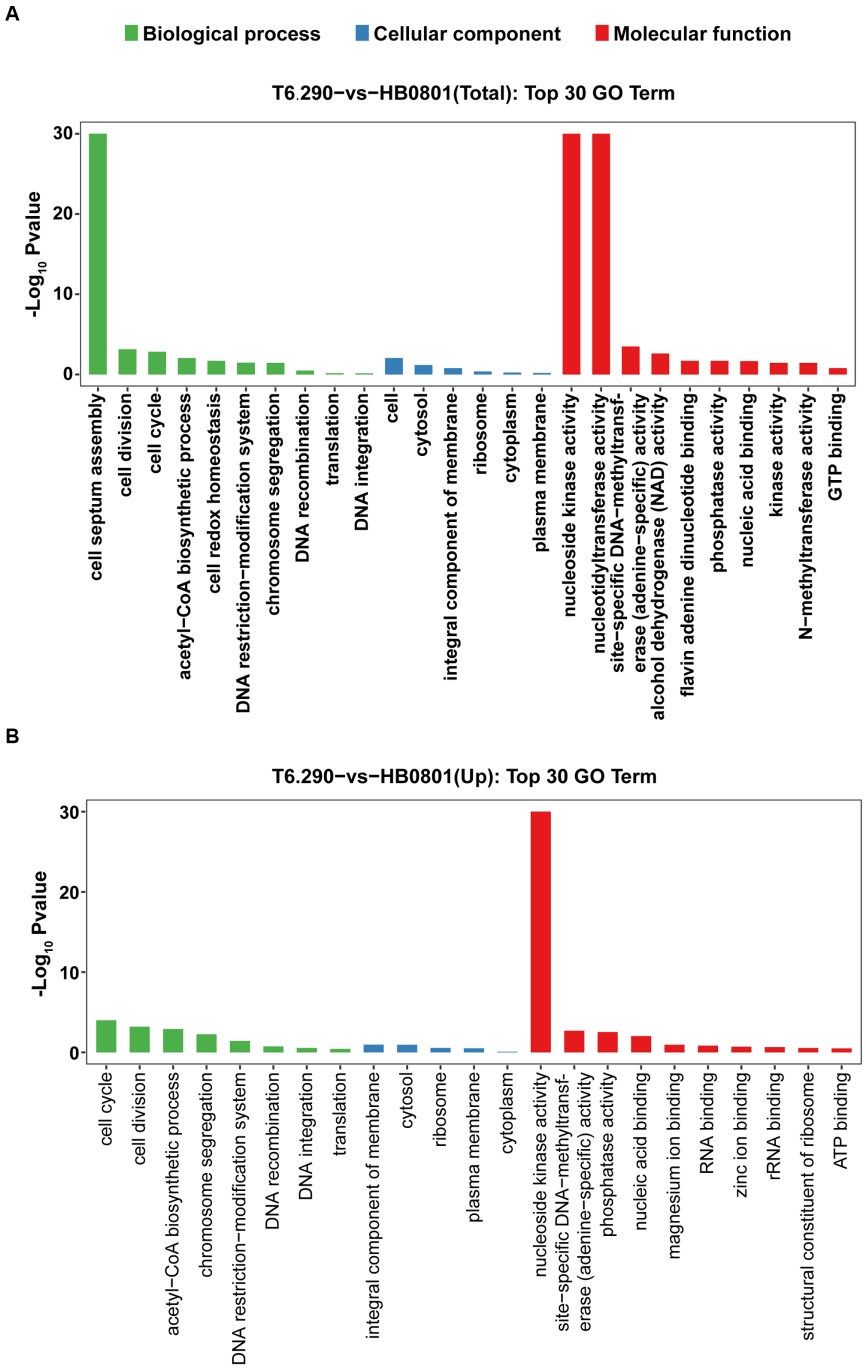
Mapping of DEGs by Gene Ontology (GO) function. The top 30 GO term enrichment analysis of total DEGs **(A)** and up-regulated mRNA **(B)** between HB0801 parental strain and T6.290 mutant.

**Figure 6:**
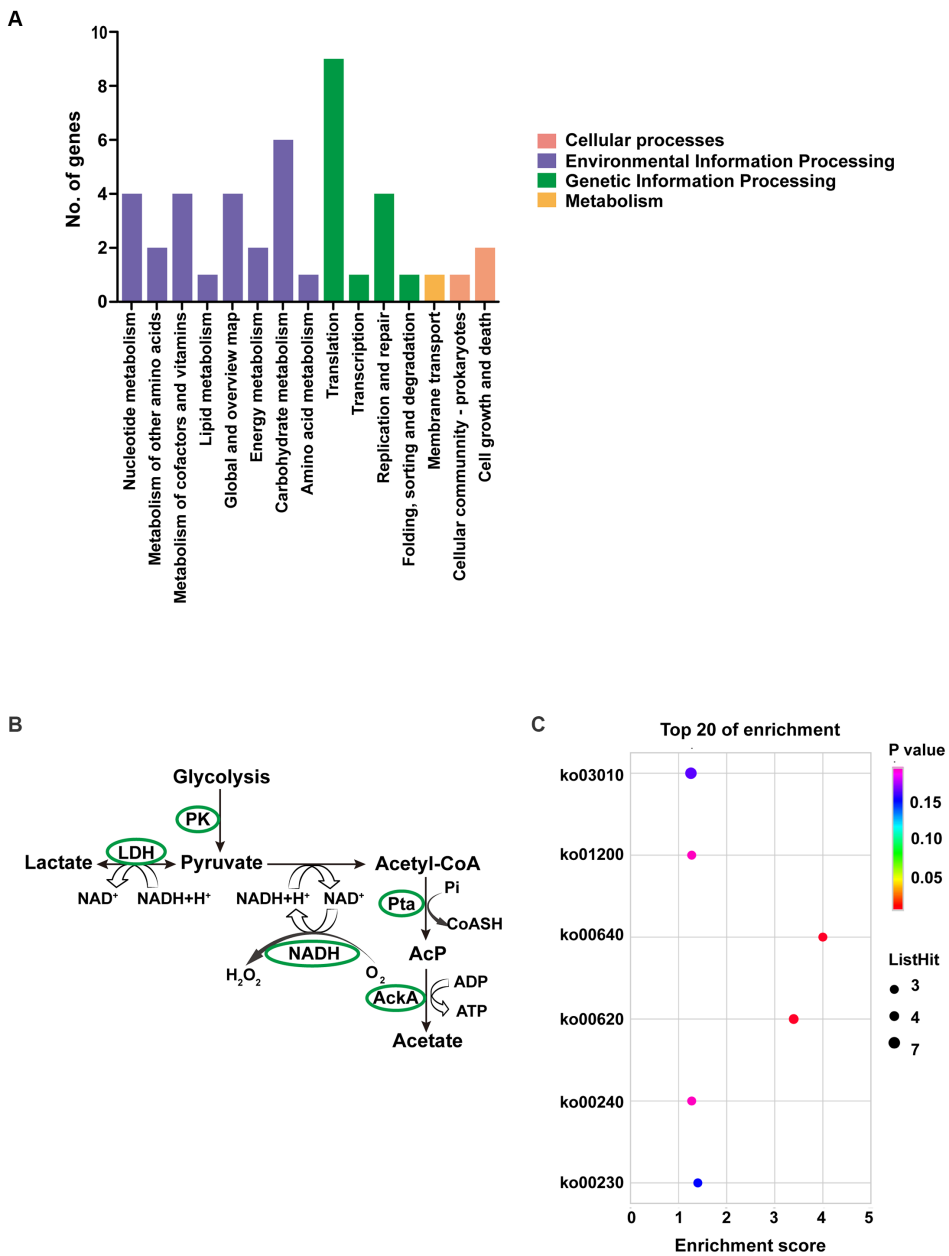
Pathway analyses of DEGs of *M. bovis* HB0801 and mutant T6.290. **(A)** KEGG pathway enrichment of DEGs. **(B)** DEGs involved in pyruvate metabolism pathway. **(C)** KEGG enrichment of top 20 metabolism pathway of DEGs.

Up to 57 DEGs in T6.290 were of unknown functions. GO enrichment was carried out to analyze the functions of the 81 remaining DEGs in T6.290. According to GO annotation, DEGs significantly enriched were involved in biological process (cell septum assembly), and molecular function (nucleoside kinase activity and nucleotidyltransferase activity) (Figure 5). KEGG pathway analysis revealed many DEGs participating in genetic information processing including translation, replication and repair (Figure 6A) and a vast group of DEGs participating in metabolism such as nucleotide, amino acid, cofactors and vitamins, as well as carbohydrate metabolism (Figure 6A). Up to 7 DEGs were involved in central metabolism including upregulated genes Mbov_0150 (Phosphate acetyltransferase, Pta), Mbov_0151 (Acetate kinase, AckA) and Mbov_0312 (Alcohol dehydrogenase, Adh), as well as down-regulated genes Mbov_0155 (Pyruvate kinase, Pk), Mbov_0160 (Lactate dehydrogenase, LdhA), Mbov_0286 (NADH oxidase (NOXASE), HcaD) and Mbov_0338 (Alcohol dehydrogenase, Adh) (Figure 6B). Among them, 4 genes (Mbov_0150, Mbov_0151, Mbov_0155 and Mbov_0160) are involved in pyruvate metabolism (Figure 6C). Indeed, genes involved in acetate production were up-regulated in the mutant, while genes that participate in the production of lactate were down-regulated. The Mbov_0286 product is the NADH oxidase (NOXASE) which is involved in the H_2_O_2_ production. These data illustrate the broad transcriptomic response of mycoplasma cells to the loss of GpdP activity and indirectly the central regulatory role played by GpdP and its secondary messenger substrates in the biology of *M. bovis*.

## 4 Discussion

Cyclic-di-AMP homeostasis is essential for bacterial growth and virulence, as both the accumulation and depletion of this signaling molecular are detrimental to the bacteria (Corrigan and Grundling, 2013; Mehne et al., 2013; Whiteley et al., 2015). The PDEs of the GdpP family play a central role in the metabolism of this second messenger (Rao et al., 2010; Du et al., 2014; Wang et al., 2017). Remarkably, MbovGdpP was found to be a multifunctional enzyme exhibiting ssDNase activity in addition to the typical PDE function associated with bacterial GdpP and the nanoRNase activity (Zhu et al., 2020). However, despite showing similarity with exonuclease RecJ, nanoRNA and c-di-AMP may be the preferred substrates of MbovGdpP, since the enzyme only displayed a limited ssDNase activity. However, a quantification of catalytic efficiencies for each substrate is needed to confirm this hypothesis. Long incubation times needed to observe ssDNA degradation raised questions regarding the origin of the ssDNase activity, with possible contamination of recombinant MbovGdpP proteins with a ssDNase from *E. coli*. However, this hypothesis was ruled out by the catalytically dead recombinant protein MbovGpdP_158–666_, which excluded any contamination with *E. coli* ssDNase. This unusual PDE activity, which was only previously reported for the ssDNA exonuclease RecJ (Han et al., 2006; Handa et al., 2009; Zhang et al., 2022), suggests a possible role of MbovGdpP in nucleotide recycling. Indeed, cyclic dinucleotide PDE and nanoRNase activities were recently found essential for nucleotide recycling in *M. bovis* (Zhu et al., 2020). Remarkably, extracellular DNA was recently identified as a nutritional limiting factor for *M. bovis* proliferation under cell culture conditions and cytotoxicity of this pathogenic species. Altogether, these results point towards nucleotide metabolism as strategic for *M. bovis* interaction with host cells (Zhu et al., 2019, 2020). Far from its classical role in the storage of genetic information, DNA is also pivotal for bacterial physiology. In several species, such as *Vibrio cholera*, *Pseudomonas aeruginosa*, *Shewanella*, *Serratia marcescens* and *S. aureus*, eDNA can be degraded into nucleotides, which are further used as phosphate, carbon and nitrogen sources (Beliaeva et al., 1976; Pinchuk et al., 2008; Gumpenberger et al., 2016; McDonough et al., 2016; Vorkapic et al., 2016; Lewenza et al., 2020). More interestingly, the extracellular DNase MbovNase and MnuA were recenly identified as a virulence factor in *M. bovis* (Zhang et al., 2016; Mitiku et al., 2018) and a key element for this pathogen to escape neutrophil extracellular traps (NETs) (Zhang et al., 2016; Mitiku et al., 2018).

The ability to withstand external pressures, such as salt and other osmotic stresses, is critical for mycoplasmas that lack a cell wall. Our results suggest that osmotic tolerance may be regulated by MbovGdpP in *M. bovis*. This result is consistent with the role of c-di-AMP specific PDEs in classical bacteria. Indeed, deletion of GdpP can lead to hypersensitivity to Na^+^ and/or K^+^ salts in several species including *Listeria monocytogenes*, *Lactococcus lactis*, *Bacillus anthracis*, *S. aureus*, and *Streptococcus gallolyticus subsp. gallolyticus* (Smith et al., 2012; Moscoso et al., 2016; Pham et al., 2018; Teh et al., 2019; Hu et al., 2020; Schwedt et al., 2023).

In *L. monocytogenes*, GdpP is linking cyclic nucleotide metabolism to osmotic tolerance by regulating intracellular c-di-AMP levels. Indeed, this secondary messenger was found to bind to several target proteins and regulate their activities (Schwedt et al., 2023). In *S. aureus*, the osmosensitive phenotype was associated with altered expression of osmotic receptors such as K^+^ and glycine-betaine transporters (Moscoso et al., 2016; Schuster et al., 2016; Zeden et al., 2018). Whether these osmotic receptors are involved in the osmotic tolerance of *M. bovis* is unknown, but the cytosolic regulatory protein KtrC, a protein found associated with Ktr ion transporters in other bacteria, is one of the c-di-AMP receptors in *M. pneumoniae* (Blotz et al., 2017).

Potassium is essential for cell survival and physiology, such as osmoregulation, pH homeostasis, regulation of protein synthesis, enzyme activation, membrane potential adjustment, and electrical signaling (Binepal et al., 2016; Stautz et al., 2021). For example, the growth of *S. mutans* is sensitive to extracellular K^+^ availability, and low or high K^+^ concentrations result in delayed bacterial growth (Binepal et al., 2016). In *B. subtilis*, the concentration of extracellular K^+^ is reported to influence biofilm formation (Fall et al., 2006; Lopez et al., 2010). In the foodborne pathogen *Salmonella enterica*, high environmental K^+^ concentrations increased the expression of virulence factors and host cell invasion (MacGilvary et al., 2019). The physiological relevance of potassium stress in *M. bovis* remains to be further investigated. Interestingly, several mycoplasma species, including *M. bovis*, have the ability to invade host cells and to survive intracellularly (van der Merwe et al., 2010; Burki et al., 2015; Mizuki et al., 2015; Niller et al., 2017; Nishi et al., 2021). Upon cell invasion, bacteria have to face important changes in potassium concentration, which vary from 4 mM K^+^ in host blood and tissue fluid to 150 mM K^+^ in the cytoplasm (Xue et al., 2011; Stautz et al., 2021). Whether MbovGdpP may facilitate cell invasion by *M. bovis* remains largely unknown.

The nucleotide ppGpp is a signaling molecule involved in the bacterial response to nutrient starvation (Chatterji and Ojha, 2001; Kalia et al., 2013). As reported, the intracellular ppGpp in bacteria can rapidly accumulate up to a millimolar level under starvation conditions (Potrykus and Cashel, 2008; Srivatsan and Wang, 2008). In several bacterial species, such as *B. subtilis*, *S. aureus* and *E. faecalis* (Rao et al., 2010; Corrigan et al., 2015; Wang et al., 2017), up to 1 mM of ppGpp was used to confirm that this molecular can inhibit the PDE activity of GdpP, which covered the concentration used in our study. Remarkably, ppGpp was found to inhibit the nanoRNase activity of MbovGdpP, but not its PDE activity. This suggests that PDE and nanoRNase activities may be mediated by different catalytic sites in MbovGdpP, and that ppGpp may competitively bind to the nanoRNase active site.

c-di-AMP-specific PDE can have an important impact on gene expression (Zarrella et al., 2020; Zhu et al., 2020). Interestingly, our study highlighted an altered expression of tRNA in the MbovGdpP knock-out mutant. In *L. monocytogenes*, lacking c-di-AMP lead to the accumulation of alarmone molecular (p)ppGpp (Peterson et al., 2020), according to the report in *E. coli* and *M. capricolum*, the accumulation of ppGpp and pppGpp would further influence the stability of tRNA (Glaser et al., 1981; Fernandez-Coll and Cashel, 2020). Thus we speculate that ppGpp may play a bridge connecting c-di-AMP and tRNA levels in *M. bovis*.

A considerable number of DEGs in the MbovGdpP knock-out mutant were associated with replication, recombination, repair and translation. These data were consistent with the association of the DHH superfamily with a broad range of cellular processes (Srivastav et al., 2019). KEGG analysis further revealed an influence of MbovGdpP on pyruvate metabolism genes and energy production (Hegde et al., 2015). The down-regulation of energy-producing genes in the MbovGdpP knock-out mutant may have important implications *in vivo*, as suggested by the growth-deficient phenotype exhibited by this mutant cell under cell culture conditions (Zhu et al., 2020).

While identifying the role of multifunctional genes in minimal bacteria, this study further illustrates the necessity of nucleotide metabolism in maintaining the normal physiological activities of mycoplasmas.

## Data availability statement

The data presented in the study are deposited in the Gene Expression Omnibus (GEO) repository, accession number GSE233141 (https://www.ncbi.nlm.nih.gov/geo/query/acc.cgi?acc=GSE233141).

## Author contributions

AG, XZ, and EB designed the study and wrote the main manuscript text. XZ, EB, ZH, XL, GZ, and YD contributed in collecting and analyzing the data. All authors contributed to the article and approved the submitted version.

## Footnotes

^1^ https://www.ncbi.nlm.nih.gov/tools/cobalt

^2^ http://espript.ibcp.fr/ESPript/ESPript/
