# Supplementary figures and images for: An atypical GdpP enzyme linking cyclic nucleotide metabolism to osmotic tolerance and gene regulation in Mycoplasma bovis
Source: Front Microbiol. 2023 Nov 30;14:1250368. doi: 10.3389/fmicb.2023.1250368 (PMC10720645; doi:10.3389/fmicb.2023.1250368)

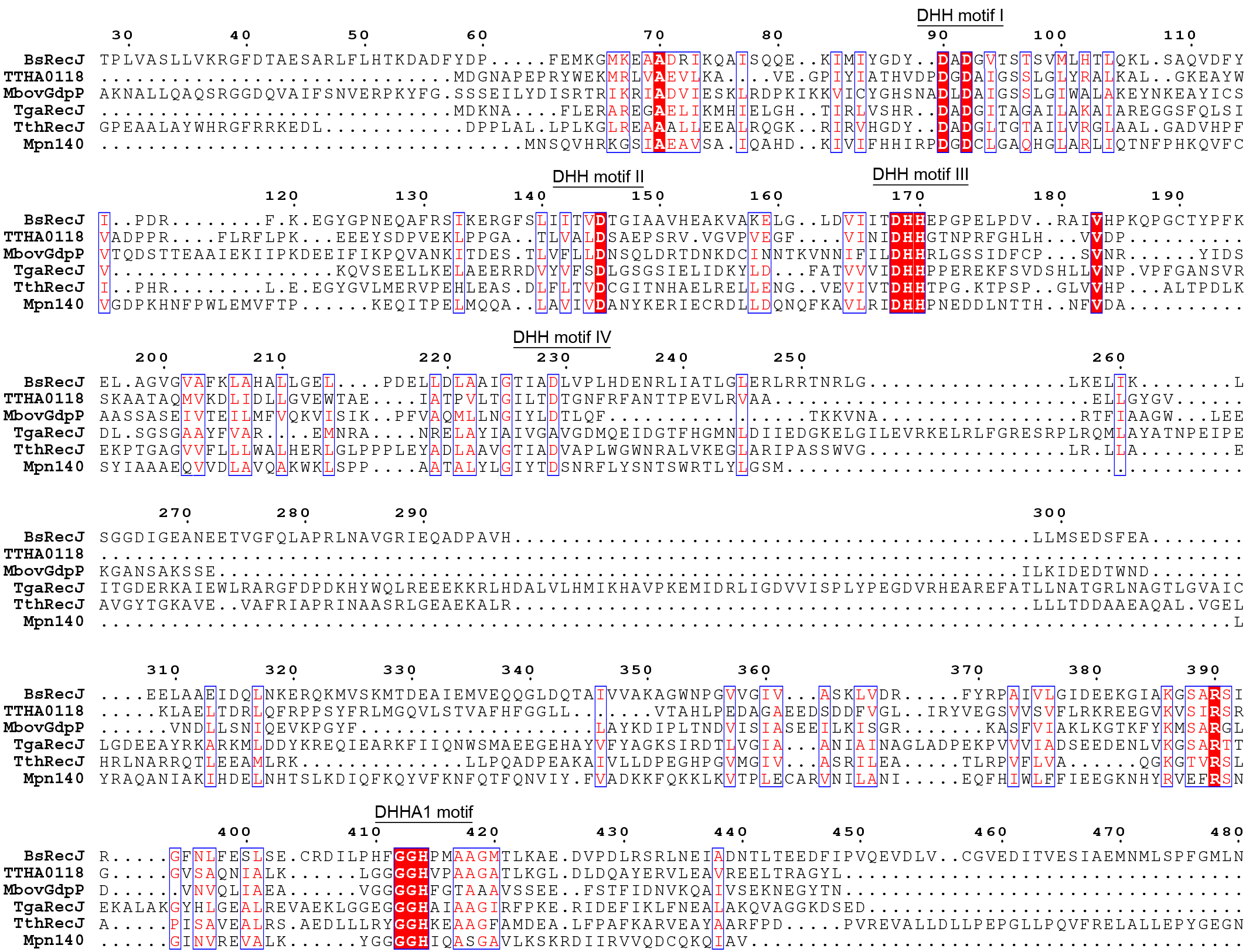

Supplement: Supplementary file 3 [file Image_1.TIF]

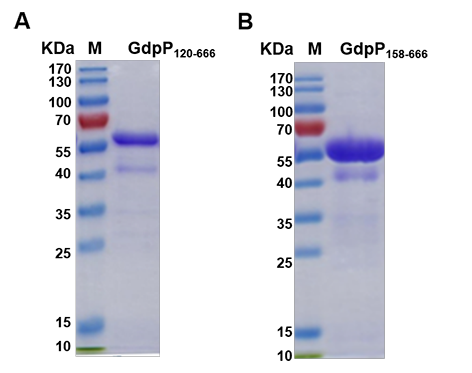

Supplement: Supplementary file 4 [file Image_2.TIF]
